# Graph-Based Analysis of Visual Scanning Patterns: A Developmental Study on Green and Normal Images

**DOI:** 10.1038/s41598-020-63951-3

**Published:** 2020-05-08

**Authors:** Padideh Yazdan-Shahmorad, Negar Sammaknejad, Fatemeh Bakouie

**Affiliations:** Institute for Cognitive and Brain Sciences, Shahid Beheshti University, Tehran, 19839-63113 Iran

**Keywords:** Neuroscience, Psychology, Mathematics and computing

## Abstract

The present study investigated the visual scanning pattern of children with typical development in three different age groups(4–6,6–8,8–10 years old). We used a data set from one related research, which included images with different low-level features: Green and Normal. This study analyzed age-associated inter-individual differences and was intended to show that graph profiling combined with a fixation time approach could help us to better understand the developmental visual pattern. Thus, degree centrality as one of the graph theory measures was implied to analyze gaze distribution. We explored the influence of bottom-up features, comparing the first 2 s (early phase) with the interval from 4 to 6 s (late phase) of scene exploration during age development. Our results indicated that degree centrality and fixation time increased with age. Furthermore, it was found that the effects of saliency are short-lived but significant. Moreover, we found that Green images during the early phase play an important role in visual anchoring, and the children’s performance was significantly different between 4–6 y and 6–8y-group. This comparative study underscores the ability of degree centrality as a developing innovative measure to perform eye-tracking data analyses.

## Introduction

Our eyes continuously scanned the environment. Their movements within the scene have classified gaze into two general subsegments: fixation and saccade^1^. During fixation, the visual scene can be analyzed by details, but only a very small part of it. Therefore the eyes have to switch swiftly to other scene regions by very fast movements named saccades^2,3^. The result of successive fixations and saccades through time and space is the visual scan path, which may overlap itself^4^.

Goal-driven attention can be described as a top-down process that is based on innermost control, such as behavioral task demands^5–8^. On the other hand, stimulus-driven attention can be described as a bottom-up process. Visual characteristics of a stimulus such as color, contrast, and movement can draw attention to the stimulus. These low-level visual features are categorized as saliency. There is extensive evidence that during the early viewing phase (0–2 seconds) of scene viewing, visual scan path mainly guided by low-level features^5–10^. Although it is still difficult to demonstrate if and when bottom-up and top-down processes can contribute to guide eye movements^3,11,12^. The timing account is a viewpoint that explains a wide range of observations^13,14^. It assumes that low-level features can be processed faster in relation to top-down control. According to this view, operation of bottom-up process starts immediately after the onset of stimulus^14–17^. The operation of top-down processes overrides bottom-up processes immediately after the early time window or might overlap it^18^. Fitting with the timing account, studies suggest that the effect of low-level features wanes within 10–20 first fixations^19^.

Another recent aspect of viewing behavior is attentional processing. It can be distinguished into two categories by fixation lengths and saccade amplitudes. According to this categorization, short fixations (<180 ms) that are followed by large amplitude saccades (>5) are grouped into a mode, called ambient mode and found mostly in the first 2 seconds of scene viewing. Also, long fixations (>180 ms) that are surrounded by saccades of short amplitudes (<5) are categorized to another one called focal mode and appear beyond the first 2 seconds^20–23^. Henceforward, it assumes that bottom-up control is more related to the ambient mode, and top-down control is rather associated with the focal mode. It seems that ambient mode is more correlated with dorsal pathways^20,23,24^, and focal mode is associated with ventral pathways^24,25^. However, the dorsal pathway matures after the ventral pathway, and this brings us to the question of whether the focal mode is more dominant in younger children compared with older ones. In this study, one of the main aims is to analyze ambient and focal attention modes during age development. In doing so, the time-course of visual scanning pattern is examined during development in three different age groups (4–6, 6–8, 8–10 years old).

Moreover, the present paper shows that graph profiling combined with the classical fixation time approach will help us to understand the development of visual patterns more precisely. The classical analysis does not provide means to trace shifts in attention due to its static nature. On the other hand, the scan path is an appropriate choice to probe its dynamic action. However, it does not give enough indices to analyze the trace^26–28^. Matsuda and Takeuchi^28,29^ filled the gap between these approaches by applying network analysis. The partially dynamic and synthetic nature of the network enables us to attribute a graph into a visual scan pattern and extract more details from it. Data about relations between Areas Of Interests (AOIs) can be summarized and represented as a set of nodes and edges, forming a graph^30^. Probing such a graph has led to derive different measures (such as degree centrality), which enables us to compare multiple visual scan paths. This method has developed by Guillon and his colleagues^31^ based on the adjacency matrix that summarizes all transitions between each possible pairs of AOIs.

With the aim of establishing a framework for a visual scan pattern, in this study, we analyzed a dataset from another related study^32^. They had examined the effect of low-level features on visual scan patterns by conducting a developmental experiment, which included Green and Normal images.

Green images were used in Sammaknejad and her colleagues^32^ experiment, which had been produced by a perceptually based algorithm for color quantization^33^. It was shown that these images reduce energy consumption by 4.25% on average on modern energy-adaptive displays. As Sammaknejad and her collaborators^32^ assumed, these images have different low-level features compared to Normal ones. Therefore, comparing the visual scan pattern of Green images with Normal ones could be helpful in order to examine the effect of low-level features on eye-movement behavior.

Within the framework of graph theory, we analyzed the visual scan pattern during development to broaden our understanding of low-level features’ effects. This study put forward to indicate in what time window low-level features may guide gaze allocation. Moreover, it provides evidence for the existence of the ambient and focal modes. To meet this end, we analyzed scene strategies between Normal and Green Images (a) during the entire scene viewing (0–10 sec), (b) during the early phase (0–2 sec), and also (c) late phase (4–6 sec) of visual scanning. Additionally, we compared two different methods: fixation time analysis as a traditional approach and degree centrality as an application of graph theory.

## Methods

### Data

**Figure 1:**
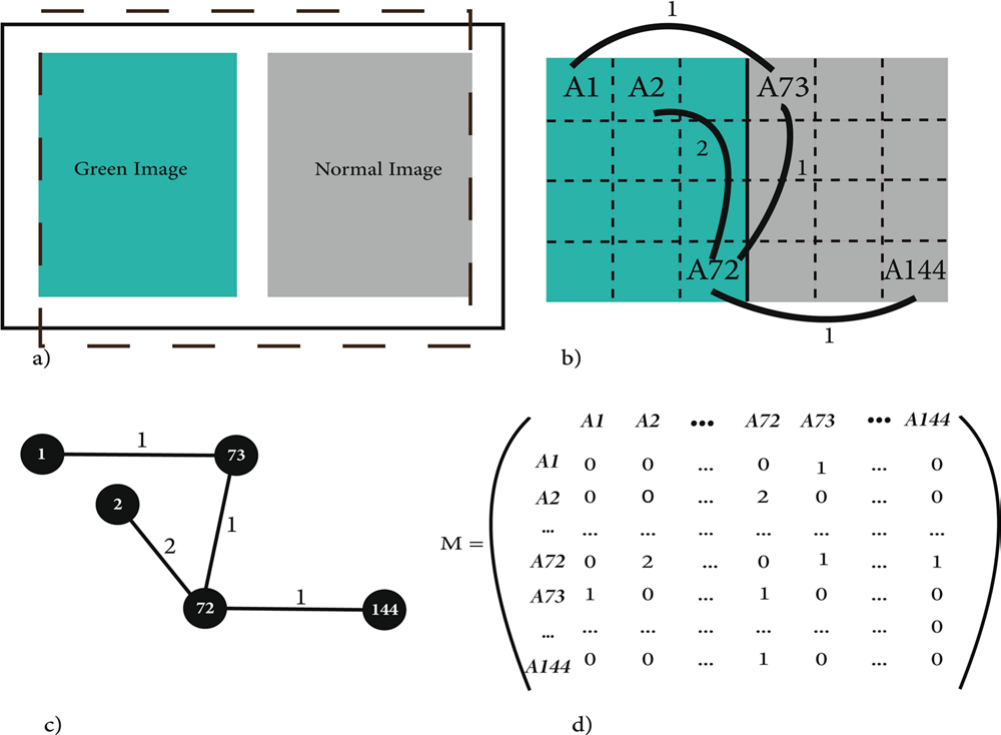
The segmentation approach on each stimulus (**a**) One stimulus consisted of a Normal and a Green image aligned together horizontally and was shown for 10 seconds. (**b**) Green and Normal as two main AOIs (Area of Interests) has been segmented into 144 AOIs by a 16 × 9 mesh. Corresponding a network to scan pattern, each AOI has been considered as one node and also fictitious links between AOIs known as edges, (**c**) The corresponding weighted undirected network with the number of links between pairs of nodes and (**d**) Weighted adjacency matrix M.

The data we used in this paper were extracted from another study^32^. They have recorded the scan patterns of 60 typically developing children from 4 to 10 years old by an SMI RED desktop eye tracking device (SensoMotoric Instruments), at the rate of 250 Hz. According to Sammaknejad and her colleagues^32^, the proposal for collecting the original data was reviewed and approved by the scientific board of the Institute for Cognitive Brain Sciences of the Shahid Beheshti University as well as the Cognitive Sciences and Technology Council of Iran, the institute that funded the original study, and the research was conducted in the institute for Cognitive Brain Sciences of the Shahid Beheshti University. The informed consent form was obtained from parents/legally authorized representatives of participants. Moreover, Helsinki guidelines were followed for this study. A five-dot calibration was performed before the experiment. The stimulus was presented in 1600 × 900-pixel resolution, 32 bit, 60 Hz on a 22-in Generic PnP monitor. During the conducted experiment, participants were asked to look freely to 48 trials; each consisted of a Normal and a Green image (taken from the Kodak color image database, with 24 lossless true color (24 bits per pixel) images of resolution 768 × 512 pixels). These images were aligned horizontally together and separated by 1 centimeter in a mid-gray colored background (Fig. 1a). Each display lasted for 10 seconds, and after that, they were asked to answer the question, “Which of the two images looks better? (Left or Right)”. The goal of the study was to evaluate the influence of low-level features of the images on the scan pattern. A BeGaze (SensoMotoric Instruments) software was utilized to extract eye movement data, which included time-stamped XY-coordinates for each participant and various events such as fixations and saccades. For the present study, we only used fixation points.

### Procedure

#### Graph Construction

We have used Graph Theory to analyze the collected eye-tracking data from Sammaknejad and her assistants^32^ study. For this reason, we segmented the visual scene to 144 Areas Of Interest by imposing a 16 × 9 mesh on the screen for each trial. In this order, each AOI covered 100 × 100-pixel resolution of the visual scene.

As described in the Material section (Fig. 1), the effective part of each visual scene consisted of two main parts, Green and Normal images. Therefore, the segments in the left image were sequentially coded by numerical labels from number A1 to A72 and in the right image were coded from A73 to A144 (Fig. 1b).

#### Adjacency Matrix

Graphs are typically visualized as node-link diagrams (Huang, 2007). In this study, graph nodes are a set of AOIs, and links correspond to transitions between AOIs. For example, Fig. 1c represents a weighted undirected graph G = (*N*, P, *w*) where nodes are *N* = 1, 2, … 144 and connections between pair of nodes are P = {(1, 73), (2, 72), (72, 73), (72, 144)} and *w* is connection weight. In this study, *w* represents the number of transitions between two AOIs. As shown in Fig. 1b, there is a transition from node A1 to node A73. Thus node A1 is adjacent to A73, and since there is only one transition between them, their connection weight is considered 1.

In order to store each graph and extract some features, we associate a matrix to it. If a graph has *N* nodes, we may associate an matrix, which is called adjacency matrix “*M*” (Fig. 1d). The connection between each two nodes is the key concept of adjacency matrix defining as follow:

1 $${M}_{ij}=\{\begin{array}{ll}w & {\rm{if}}\,{N}_{i}\,{\rm{is}}\,{\rm{adjacent}}\,{\rm{to}}\,{N}_{j}\\ 0 & {\rm{otherwise}}\end{array}$$

According to Eq. (1), when node(i) is adjacent to node(j), then node(j) is also adjacent to node(i). Therefore, *M* is symmetrical about the main diagonal (Fig. 1d).

#### Graph analysis

In the graph analysis framework, one of the well-known concepts is analyzing node importance, and it computes in three ways: degree, betweenness, and closeness (see Freeman, 1979, for the definitions). However, in the present study, we used degree centrality. According to Matsuda, Noriyuki, and Haruhiko Takeuchi^29^, “The greater the centrality of the node, the higher is its importance”.

In this study the degree centrality for each AOI (each node) has been calculated based on Opsahl and his colleagues^34^ approach:

2 $${C}_{D}^{W}(i)=\frac{{C}_{D}(i)}{max{C}_{D}}\times \frac{w(i)}{maxW}\times 100$$

In this formula, the degree centrality of a node(i) is defined as C_D_(i), and the number of links to this node is indicated by *w*(i). Opsahl^34^ had added two tuning parameters to this formula: the maximum observed degree for a node (max C_D_) and the maximum number of observed links for a focal node (max *w*). It should be noted that based on Eq.(2), the measure has been standardized, and the value of each node degree centrality ranges between 0 and 100. This enables us to compare different node degrees among trials and also participants^31^.

The sum of all the nodes (144 nodes) degree centrality was calculated and assigned to each trials’ degree centrality. For each participant, the average of all trials (48 trials) degree centrality was computed and considered as participant’s degree centrality.

#### Fixation Time

According to the segments, the spatial coordination of fixation points was mapped onto the AOIs. Fixation time spent in each AOI was calculated, and the sum of fixation time of 144 AOIs corresponded to each trial. Following this trend, for each participant, the average of 48 trials was computed. In this way, we were able to compare fixation time and degree centrality.

#### Time analysis

Since empirical evidence has revealed possible influences of time course on gaze behaviors, we analyzed fixation times and degree centrality during early and late phases of scene viewing. Based on some previous studies on children^35^ and adults^21,36^, the time interval 0–2 s has been applied as the early viewing phase, while the late viewing phase covered the 4–6 s time interval. Assuming the interval between the two phases as a gradual transition, we disregarded the 2–4 s time interval in our study. Our main interest was to examine whether the influence of low-level features is similar for all age groups. To meet this end, we applied time windows in three different ways: 1. Into the whole screen in general(we refer to this state as General in this paper), 2. Into Green images and 3. Into Normal ones.

## Results

Our goal is to analyze how low-level features affect the visual scan pattern, using graph theory. This framework, as a developing innovative measure, can help users to reveal new properties of the scanning strategies employed by children during age development. We presented the results based on fixation time and graph analysis.

### Fixation Analysis

Human Visual System (HVS) cannot sense changes below the Just-Noticeable-Difference (JND) threshold. These visual characteristics of the stimulus, such as color and contrast, are known as low-level features^37^. Using the JND model^38^, Hadizadeh and his coworkers^33^ substituted the color of each pixel in Normal images with a color that consumes less energy and is perceptually indistinguishable from the original color. Repeating this process for all of the pixels, Hadizadeh and his colleagues produced the “Green” version of the Normal image. Compared to conventional color quantization, this perceptually-based algorithm reduces the energy by an average of (4.25%) and improves the contrast by an average of (13.25%).

Sammaknejad and her colleagues^32^ showed that unlike some previous studies^35^, fixation time (F(2,58) = 4.17, p = 0.021) and fixation count (F(2,58) = 21.76, p < 0.001) increased with age. Moreover, they revealed that fixation time (F = 0.14) and fixation count (F = 2.64) were comparatively even between Green and Normal images.

#### Time Course Analysis for General State

**Figure 2:**
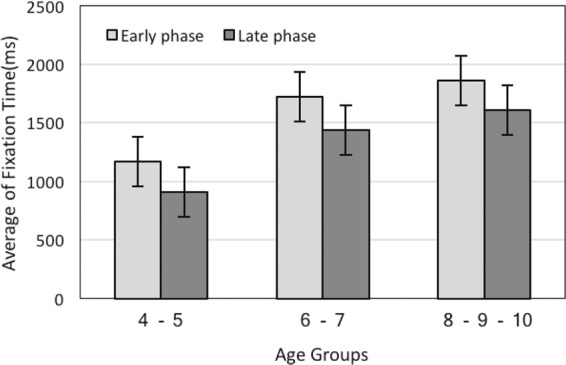
Average fixation times during the early and the late viewing phases for different age groups.

In order to analyze possible influences of the time course, we compared the average of fixation time in the early and the late phase in general (when Green and Normal images were aligned together). It should be noted that those fixations began within either of the two viewing phases but had a longer duration than the time courses were also considered for analysis. Our results showed that there is a positive correlation between the average fixation time and age in both (the early and the late) phases, revealing that the average fixation time increased with age. The correlation coefficient for the early phase was r1 = 0.93, p < 0.001 and for the late phase was r2 = 0.92, p < 0.001(Fig. 2).

The average fixation time was examined for differences along the time course by conducting a 3 (age groups)× 2(viewing phases) repeated measures ANOVA. Statistical analysis revealed significant effects between the two phases F(1, 117) = 21.74, p < 0.001. This shows that the average fixation time reduces from the early to the late phase. Also ANOVA test along with correlation results shows significant effect for age groups, F(2, 117) = 51.15, p < 0.001. Moreover, there was no interaction between the processing phases and age groups. Therefore, the decrease in the average fixation time from the early to the late phase can be considered as a stable effect across different age groups (4–5y-group, Ms = 1170.83 vs. 906.66; 6–7y-group, Ms = 1722.41 vs. 1441.32; and 8–10y-group, Ms = 1859.31 vs. 1609.45; see Fig. 2). Additionally, posthoc pairwise t-tests revealed significant differences for the average fixation time between the phases for 4–6y-group and 6–8y-group, t(30) = −3.92, p < 0.001.

#### Time Course Analysis for Green and Normal Images

**Figure 3:**
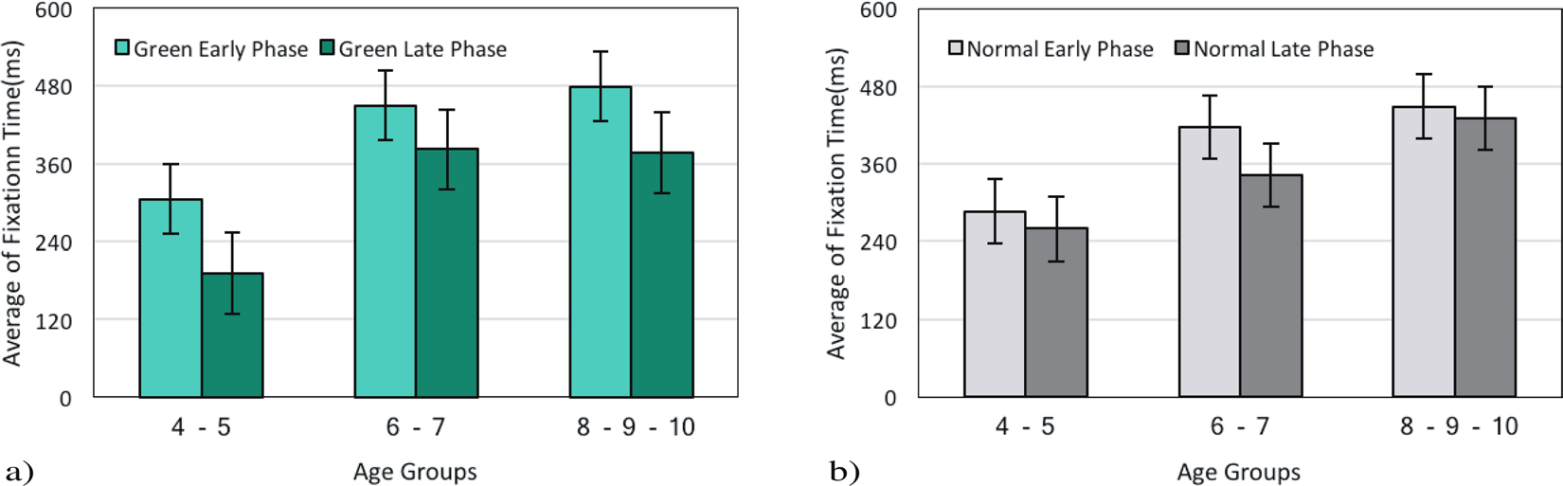
Average fixation times during the early and the late viewing phases for different age groups for (**a**) Green and (**b**) Normal Image.

The average fixation time in both phases has been calculated for Green and Normal images during age development (Fig. 3). Firstly, we compared the early and late phases of each image during age development. Then, the early phase of Green and Normal images were compared together as well as the late phase.

While analyzing Green images, we found a positive correlation between age and the early phase r1 = 0.90, p < 0.001 as well as the late phase r2 = 0.83, p < 0.001 (Fig. 3a). This result shows that the average fixation time increased with age. We also conducted the same described repeated measures ANOVA as the previous session for statistical tests. Significant effects were found for fixation time reduction from the early to the late phase, F(1,117) = 19.34, p < 0.001. Likewise, significant effects were found for an increase in fixation time with age groups, F(2, 117) = 25.66, p < 0.001. Results showed no interaction between age groups and the viewing phases: in all the groups fixation times decreased from the early to the late phase (4–6y-group, Ms = 304.94 vs. 191.84; 6–8y-group, Ms = 449.90 vs. 381.75; and 8–10y-group, Ms = 479.19 vs. 376.21; see Fig. 3a). A posthoc pairwise t-test revealed a significant difference for the fixation time between the early phase in 4–6y-group and 6–8y-group t(30) = −3.02, p = 0.005 as well as the late phase t(30) = −4.98, p < 0.001.

Examining Normal images also confirmed that the average fixation time increased with age by revealing a positive correlation between age and the early phase r1 = 0.90, p < 0.001 and the late phase r2 = 0.85, p < 0.001 (Fig. 3b). ANOVA tests disclosed significant effects for age groups, F(2, 117) = 20.14, p < 0.001; but no significant effects were found between the two phases F(1,117) = 3.42, p = 0.067 (4–6y-group, Ms = 286.03 vs. 259.51; 6–8y-group, Ms = 416.70 vs. 342.13; and 8–10y-group, Ms = 448.41 vs. 430.20), and there was no interaction, F < 1. Also for 4–6y-group and 6–8y-group more significant differences for the average fixation time during the early phase t(30) = −3.33, p = 0.002 was revealed by post-hoc pairwise t-test.

**Figure 4:**
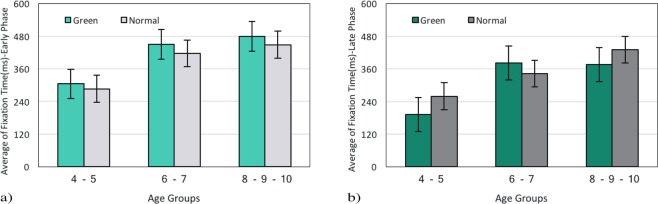
The comparison of the average fixation time between Green and Normal images in general state for (**a**) The Early Phase and (**b**) The Late Phase.

During the early phase, as shown in the Fig. 4a, the average fixation time of Normal (M(N1)) and Green images (M(G1)) are comparatively even in three age groups, F(1,117) = 2.03, p = 0.15(4–6y-group, M(G1) = 304.94 vs. M(N1) = 286.03; 6–8y-group, M(G1) = 449.90 vs. M(N1) = 416.70; and 8–10y-group, M(G1) = 479.18 vs. M(N1) = 448.42). In both Green and Normal images, the average fixation time was increased with age during the early phase, F(2,117) = 26.18, p < 0.001.

The late phase comparison of fixation time revealed relatively even average fixation time between Green and Normal images F(1,117) = 1.36, p = 0.25 (4–6y-group, M(G2) = 191.85 vs. M(N2) = 259.51; 6–8y-group, M(G2) = 381.75 vs. M(N2) = 342.12; and 8–10y-group, M(G2) = 376.21 vs. M(N2) = 430.20; see Fig. 4b). Moreover, the average fixation time of the late phase was increased during age development in both Green and Normal images F(2,117) = 19.53, p < 0.001.

### Graph Analysis

As it was mentioned earlier, our main interest was to use one of the graph theory features, the degree centrality, to analyze participants’ scanning pattern during age development and also during scene viewing.

#### Degree Centrality during Development

**Figure 5:**
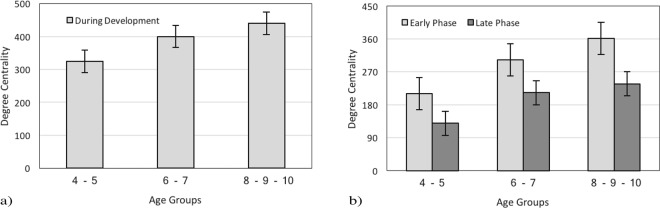
Average degree Centrality (**a**) during development and (**b**) during the early and the late viewing phases for different age groups.

Based on Opsahl and his colleagues^34^ approach, we have calculated the average degree centrality of all 144 segments during development (Fig. 5a). Degree centrality was increased during age development, and there was a positive correlation between the average degree of centrality and age r = 0.93, p < 0.001.

#### Time Course Analysis in General State

The average degree of centrality was compared during the early and late phases (Fig. 5b). The calculation revealed that there is a positive correlation between the averaged degree centrality in the early phase r1 = 0.9433, p < 0.001 and the late phase r2 = 0.9073, p < 0.001 with age. By conducting a 3 (group)× 2 (viewing phase) repeated measures ANOVA, degree centrality has been calculated along the time course. Statistical analysis showed that there was a reduction from the early to the late phase by revealing a significant difference between the processing phases, F(1, 117) = 105.61, p < 0.001 (4–6y-group, Ms = 324.25; 6–8y-group, Ms = 400.15; and 8–10y-group, Ms = 440.01; see Fig. 5b); Moreover, significant effects were found for age groups, which means degree centrality for both phases increases with age F(2, 117) = 62, p < 0.001. The decrease in the average degree of centrality from the early to the late phase could be considered as a stable effect across different age groups. There was no significant interaction between both the phases and age F(1,117) = 2.19,p = 0.12 (4–6y-group, Ms = 210.52 vs. 129.25; 6–8y-group, Ms = 302.19 vs. 213.09; and 8–10y-group, Ms = 361.50 vs. 237.55; see Fig. 5b). Post-hoc pairwise t-tests revealed significant differences for the average degree centrality between the early phase for the 4–6y-group and the 6–8y-group t(30) = −3.84, p < 0.001 as well as the late phase t(30) = −4.68, p < 0.001.

#### Green and Normal Images during Development

**Figure 6:**
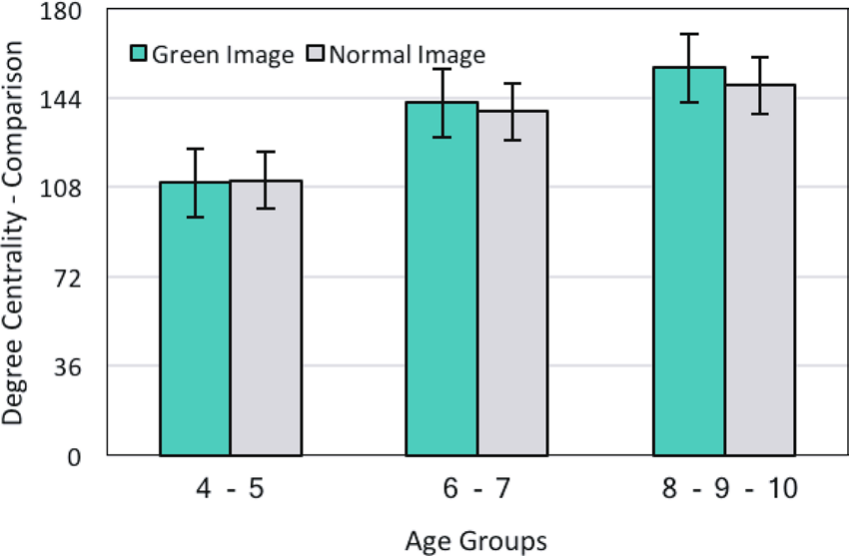
The comparison of average degree centrality between Green and Normal images.

One of the most important aspects of this study is the comparison of degree centrality between Normal and Green images. This would able us to assess the long-term effect of low-level features on the scan pattern. As Fig. 6 illustrates there is no significant difference between Green (G) and Normal(N) images F(1, 117) = 0.44, p = 0.51 and their degree centralities are comparatively even (4–6y-group, M(G) = 109.74 vs M(N) = 110.84; 6–8y-group, M(G) = 141.97 vs M(N) = 138.51; and 8–10y-group, M(G) = 117.0 vs M(N) = 149.0). However, statistical analysis revealed a positive correlation between the average degree of centrality in both images with age, F(2, 117) = 26.99, p < 0.001. In addition, there was a positive correlation between the average degree centrality and age in both Green and Normal images, r(G) = 0.92, p < 0.001 and r(N) = 0.91, p < 0.001. Therefore, the average degree of centrality in both images (Normal and Green) increases with age.

#### Time Course Analysis of Degree Centrality for Green and Normal Images

**Figure 7:**
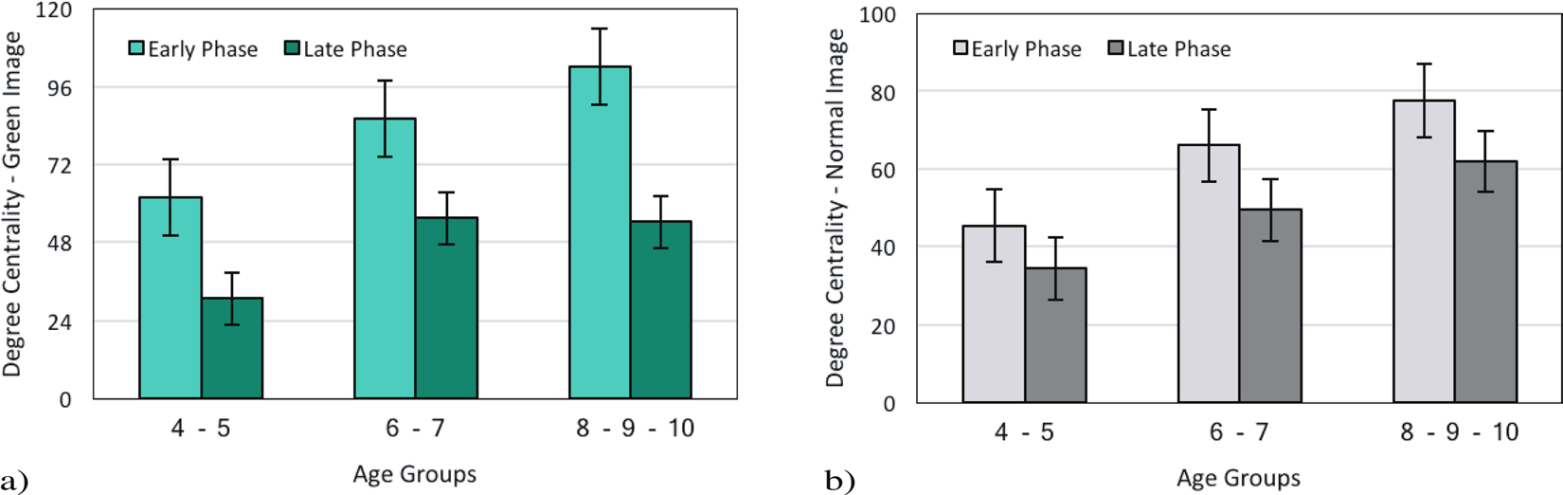
Average degree centrality during the early and the late viewing phases for different age groups (**a**) Green Image and (**b**) Normal Image.

In this part, we compared the average degree of centrality in both phases for Green and Normal images. Analyzing Green images, we found a positive correlation between the average degree centrality and age in the early phase (G1) = 0.92, p < 0.001 and the late phase r(G2) = 0.82, p < 0.001; revealing that the average degree centrality in Green images increases with age. Statistical analysis revealed that the average degree of centrality in Green images decreases from the early to the late phase F(1, 117) = 111.86, p < 0.001(Fig. 7a). Furthermore, a significant difference was found between the early and the late phases for all age groups F(2, 117) = 29.01, p < 0.001. There is a significant interaction between the both phases and age F(2, 117) = 3.2, p = 0.04 (4–6y-group, M = 61.88 vs. 30.76; 6–8y-group, M = 86.20 vs. 55.44; and 8–10y-group, M = 102.13 vs. 54.26). More significant differences were found in the average degree centrality of Green images between the early phase t(30) = −2.80, p = 0.009 and the late phase t(30) = −4.47, p < 0.001 for 4–6y-group and 6–8y-group by posthoc pairwise t-tests.

It was shown that there is a positive correlation between age and degree centrality for Normal images in the early phase r(N1) = 0.90, p < 0.001 as well as the late phase r(N2) = 0.86, p < 0.001. Moreover, there was a significant effect of degree centrality F(2, 117) = 56.06, p < 0.001 as well as processing phases F(1,117) = 7.94, p = 0.005 (Fig. 7b) in three age groups. The decrease in the average degree centrality from the early to the late phase can be considered as a stable effect across different age groups (4–6y-group, M = 45.41 vs. 34.45; 6–8y-group, M = 66.01 vs. 49.40; and 8–10y-group, M = 77.46 vs. 61.89; see Fig. 7b), but there was no interaction between processing phases and age groups, F < 1. Also, the posthoc pairwise t-test revealed significant differences in average degree centrality between 4–6y-group and 6–8y-group in Normal images during the early phase t(30) = −3.46, p = 0.002 as well as the late phase t(30) = −2.46, p = 0.020.

**Figure 8:**
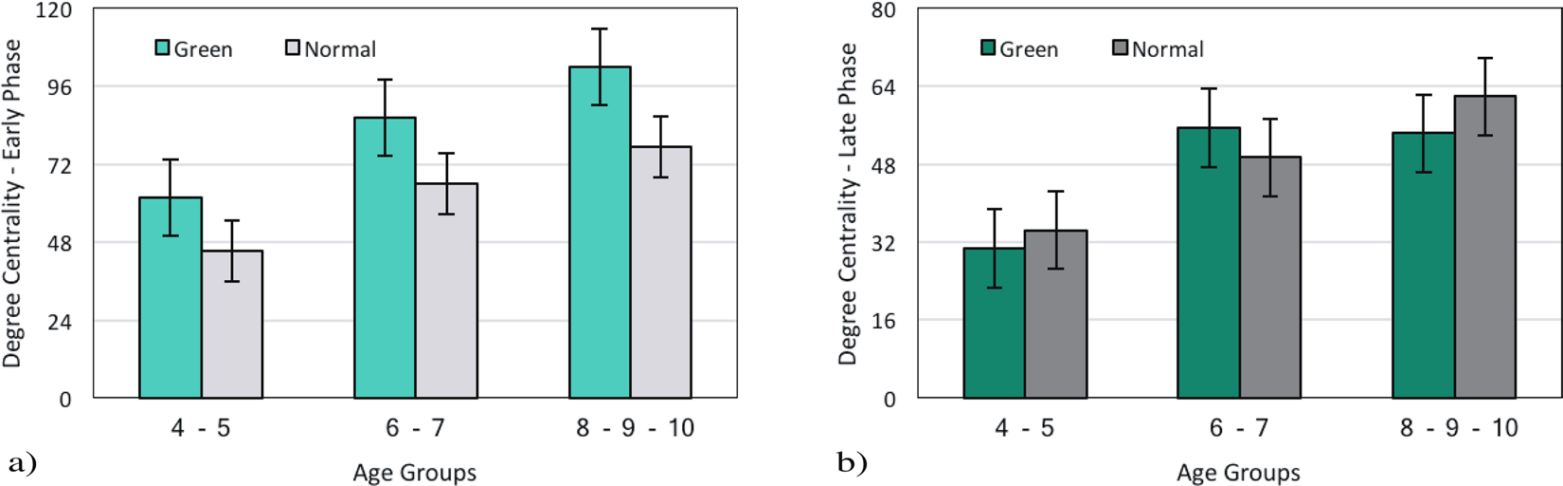
The comparison of average degree centrality between Green and Normal images in general state for (**a**) the Early Phase and (**b**) the Late phase.

In this section, we compared degree centrality between Green and Normal images in the early phase (see Fig. 8a) and the late phase (see Fig. 8b). During the early phase, the average degree centrality in Normal images are significantly lower compared with Green ones, F(1, 117) = 35.91, p < 0.001 and it is a stable effect among all age groups (4–6y-group, MG1 = 61.88 vs. MN1 = 45.41; 6–8y-group, MG1 = 86.2 vs. MN1 = 66.01; and 8–10y-group, MG1 = 102.13 vs. MN1 = 77.46). In addition, it should be noted that the average degree of centrality in the early phase has been increased during development F(2,117) = 37.8, p < 0.001.

In order to examine whether low-level features have long-term effect on eye movements, the late phase was also investigated. As seen in Fig. 8b the average degree centrality in Normal and Green images are comparatively even during late phase F(1, 117) = 0.31, p = 0.58 (4–6y-group, MG2 = 30.76 vs. MN2 = 34.45; 6–8y-group, MG2 = 55.44 vs. MN2 = 49.40; and 8–10y-group, MG2 = 54.26 vs. MN2 = 61.89). Also, the average degree centrality in the late phase has been increased during age development F(2,117) = 22.28, p < 0.001.

## Discussion

In the present paper, we conducted a comparative study to capture more details of visual scanning patterns during age development. In doing so, we compared degree centrality evaluation based on graph theory and fixation time analysis (as a traditional approach). Moreover, this study attempts to clarify what time window low-level features may guide gaze allocation. Therefore, the existence of long-term effects of these features has been probed during (a) entire scene viewing (10 s), (b) early phase (0–2 s), and (c) late phase (4–6 s) of scene viewing. The present paper also puts forward to provide evidence for the existence of the two distinct processing mechanisms: (a) ambient and (b) focal mode.

### Degree Centrality of Green and Normal images

Considering that Green images have different low-level features compared with Normal ones^33^, we expected different scanning patterns while viewing the stimuli. However, our graph analysis revealed that while scanning the entire scene, there is no significant difference in the average degree of centrality between Green and Normal images in each age group (see Fig. 6, section 3.2.3). This finding is along with Sammaknejad and her colleagues^32^ results on fixation time and fixation count. They discussed that the effect of saliency is short-lived and does not last for 10 seconds (i.e., the entire scene viewing). Therefore, we conducted a further evaluation, applying a time-course analysis.

### Degree Centrality during Development

We calculated degree centrality for the entire scene, Green and Normal images together, and found that it increases with age (see Fig. 5a, section 3.2.1). This finding is in line with Sammaknejad and her colleagues^32^ analysis based on fixation time and fixation count. Although the results are not similar to those of Helo and his colleagues^35^ at first glance, the varying results is perhaps due to the design of the experiment. Here, Normal images were shown with Green images, which, according to Hadizadeh’s work^33^, consume less energy (4.25%). Thus, the presence of Green images may cause a stronger impact of saliency for older children (8–10 y-group) than younger ones. We suggest that the increase in degree centrality with age might be associated with the maturation of the visual pathway, occurring around 6 years of age^39–42^. This means that we might be able to link the maturation of eye movement with low-level features of the stimulus: as children grow up, their visual system probably learns to consume less energy due to the maturation of visual pathways.

### Time Course Analysis in General State

According to the timing account view, the operation of bottom-up control, which processes low-level features, starts immediately after the onset of the scene^14–17^. While the majority of attentional studies showed a fast decaying effect of low-level features on eye movement behavior^15,16,18,43^ others suggested a slower decay in the effect of these features^10,13,35,44,45^. Henceforward, we compared the early (0–2secs) and the late phase (4–6secs) to question the influence of low-level features in different time windows. We found greater fixation time (see Fig. 2, section 3.1.1) and degree centrality, (see Fig. 5b, section 5.2.2) in the early phase rather than the late phase during age development. This outcome indicates that there is a difference between the two-time windows and also highlighted the reduction from the early to the late phase. The underlined difference is along with earlier studies^3,14–16,44,46^ suggesting that low-level features may influence the visual scan pattern, but only in a limited time window, the early phase.

The decrease from the early phase to the late phase contrasts with some previous studies^35^. A possible explanation for this difference might be the design of the task. Due to the experiment question, “which of the two images looks better? (Left or Right)” participants were not looking for particular information in the pictures. In other words, the task did not involve them in any difficult cognitive processes. Therefore, we observed a decrease in fixation time and degree centrality as time passes, possibly because of the essence of the task. This is in line with the hypothesis that the nature of the task is directly related to the scanning pattern^3,5–8,35^. Since scene explorations might provoke different patterns of eye-movements^7^ this assumption could be investigated with more details by different tasks and varying difficulty in future work. Our results, however, do not allow answering this question, due to Sammaknejad and her collaborators^32^ experiment design.

### Time Course Analysis in Green and Normal Images

In order to capture the influence of low-level features more precisely, we compared the early and the late phase during the exploration of Green and Normal images. Based on previous studies, we expected that Normal images interest less attention during the early phase rather than Green images; since these images consume less energy (4.25%) than Normal ones^33^. The traditional approach, fixation time, illustrates no difference between Green and Normal images during the early phase (see Fig. 4a, section 3.1.2). However, our analysis clarifies a significant difference in degree centrality between these pictures in the early phase (see Fig. 8a, section 3.2.4). This shows that the degree centrality approach benefits us to investigate visual scanning patterns more accurately. This finding might also suggest the exhibition of exploratory eye movement in this phase. Moreover, our results along with several studies have shown that exploratory eye movement is associated with age (see Fig. 2, section 3.1.1; Fig. 3, section 3.1.2; see Fig. 5a, section 3.2.1; see Fig. 5b, section 3.2.2; see Fig. 7, section 3.2.4)^47–49^.

Comparing the late phase of both pictures, it is found that the average fixation time (see Fig. 4b, section 3.1.2) as well as the average degree centrality (see Fig. 8b, section 3.2.4) were relatively even between Green and Normal images. This suggests that low-level features of Green images have not influenced visual scan patterns during the late phase.

Following this trend, for both Green and Normal images, we compared fixation time during the early and the late phase in each image (see Fig. 3, section 3.1.2). This evaluation has been done for degree centrality as well(see Fig. 7, section 3.2.4). Examining Green images revealed that during the early phase fixation time and degree centrality were greater than the late phase. However, the traditional analysis did not reveal the difference between the two phases in Normal images. Along with previous studies^31^, the present paper has also highlighted the greater flexibility of the degree centrality method to reveal more details from the visual scanning pattern. Within the graph theory framework, an AOI could be considered as an anchor of the visual scanning pattern if it has a high degree of centrality^31^. In the present study, the degree centrality approach demonstrated that a Green image is an anchor in the visual scanning pattern during the early phase in typically developing children from the age of 4 to 10 years.

### Ambient and focal modes

Applying these time windows on the visual scanning pattern also has provided further support for the distinction between ambient and focal visual processing. It seems that bottom-up control during the early phase is more associated with ambient mode, which is related to pre-attentive scanning with the exploration of the spatial layout. Along with previous studies^20–23,50^ our results implied dominancy of ambient mode during the early phase by presenting longer fixation time and greater degree centrality for Green images (see Fig. 3a, section 3.1.2; see Fig. 7a, section 3.2.4) and also in General state (see Fig. 2, section 3.1.1; see Fig. 5b, section 3.2.2). In other words, it shows that during the very first seconds, the spatial distribution is guided by low-level features of the image. In addition, during the late phases of scene exploration, top-down control is more associated with focal mode, which was found to be related to attentive processing and identification of object features^5,7,8,22,23,35,50^. In line with previous studies^35^, this finding suggests that the ambient and focal processing modes already exist at the age of 4 years.

With reference to previous investigations^20,23–25,40–42,50^ the ambient mode is known to be related to dorsal visual pathways rapidly transferring visual information with low spatial resolution. It has shown that the function of dorsal visual pathways might mature above 8 years of age. The focal mode is related to the activity of ventral visual pathways, and it is found that some associated functions of this pathway might mature around the age of 6 years^24,25^. As we have mentioned earlier, the average fixation time and degree centrality is greater during the early phase rather than the late phase (except the average fixation time in Normal images) and is a stable effect among all three groups (see Fig. 3a, section 3.1.2, see Fig. 5b, section 3.2.2; see Fig. 7, section 3.2.4). We suggest that the dominance of ambient mode in older children might be related to the earlier maturation of the ventral visual pathway. This finding is in accordance with previous studies^40–42^.

Further analysis showed that there were significant differences in fixation time and degree centrality during the early and late phases between the 4–6y-group and the 6–8y-group. The greater fixation time and degree centrality during the early phase in 6–8y-group children suggest a dominance of ambient mode in these children. Moreover, It has been shown that the viewing behavior of children becomes adult-like around 10 years^51–54^. As discussed by Helo and his colleagues^35^ the dominancy of focal mode in younger groups might be related to the earlier maturation of the ventral visual pathway. The difference between these age groups has been observed in different kinds of studies related to local and global processing. They found a transition in visual preference occurring around 6 years of age^55^. As demonstrated in work by Poirel and his colleagues^39^ this shift from local to global preference is related to an anatomical maturation of the brain areas associated with the dorsal pathway. Therefore, we suggest that the significant differences in the average fixation time and degree centrality between the early and the late phases for the 4–6y-group and the 6–8y-group, may be associated with their brain anatomical findings as well (see Fig. 3, section 3.1.2; see Fig. 7, section 3.2.4).

### Age and time windows interaction

Our results illustrate a significant interaction in degree centrality between age and time course in Green images (see Fig. 7, section 3.2.4). It means that both factors (age and time courses) guide eye movement. This outcome suggests that during the early phase, bottom-up features dominate the spatial distribution as children grow up. As Aç1k and his colleagues^56^ discussed, our visual scene is guided by bottom-up features more strongly during childhood, and top-down strategies become more prominent during age development. Moreover, it was shown that the influence of top-down processes overrides the bottom-up processes as time passes^18^. Our analysis confirmed this by indicating the decrease in degree centrality from the early to the late phase. This finding proves that the degree of centrality analysis benefits us to achieve a combination of breadth and depth of coverage and detail in a visual scanning pattern.

## Conclusion

Conducting a comparative study, our results indicated age-related differences in visual scan patterns and showed that the effects of saliency are short-lived but significant. While fixation time evaluation does not bear to disclose visual scan patterns more precisely, the degree centrality approach provides us a great prediction of gaze distribution. Therefore, our study highlights the importance of degree centrality as a developing innovative graph theory-based measure to perform eye-tracking data analyses.

There are lots of studies that deal with the definition of the early and late phases. Although a number of questions in this field still remains. As Helo and his colleagues^35^ discussed, one of the important issues is the boundaries of time windows and its application in different age groups. Based on previous investigations^35^, we considered 0–2 seconds as the early phase and 4–6 seconds as the late phase across all different age groups, but the more exact mechanism of the phase classification remains unclear and could be studied in future work.
